# Network study of the nutritional patterns, the metabolic and the psychological status among overweight-obese young adults

**DOI:** 10.3389/fnut.2025.1666688

**Published:** 2025-09-19

**Authors:** Geovany Genaro Reivan Ortiz, Roser Granero, Daniel Icaza, Laura Maraver-Capdevila

**Affiliations:** ^1^Catholic University of Cuenca, Cuenca, Ecuador; ^2^Department of Psychobiology and Methodology, Universitat Autònoma de Barcelona, Barcelona, Spain

**Keywords:** overweight, obesity, nutrient patterns, cardiometabolic, anthropometric, body mass index, HOMA, network

## Abstract

**Background:**

The classification of severely overweight individuals as “metabolically unhealthy obese” (MUO) versus “metabolically healthy obese” (MHO) is based on the presence or absence of cardio-metabolic risk factors, respectively. However, evidence is limited for the differences in the underlying relationships between nutritional habits, physical measures and psychological features. This study applies network analysis to visualize the interrelationships between nutritional dietary patterns, physical measures and psychological variables in young overweight or obese adults. In addition, we identified the nodes with the highest centrality indexes, and explored their empirical modularity. Stratified networks were obtained separately for MHO versus MUO subsamples, in order to explore differences in metabolic status.

**Methods:**

Data were collected from *N* = 188 young overweight or obese adults (university students, men and women aged 18 and 25 years) and subsequently analyzed.

**Results:**

In the MHO group, stress was identified as the bridge node, with the strongest connections with other psychological features (depression and anxiety), physical measures (body mass index, triacylglycerol, hypertension and glucose) and a nutritional pattern characterized by the intake of carbohydrates, fat and sodium. In contrast, in the MUO group, a nutritional pattern characterized by high consumption of fats and sodium, closely followed by cholesterol levels, emerged as the bridge node, with strong links to other dietary habits and variables.

**Conclusion:**

Identification of the most influential nodes among young individuals with and without cardio-metabolic risk factors provides valuable insights for the design of targeted treatment strategies. A combination of classical approaches (such as change in diet, physical activity, anti-obesity drugs and bariatric surgery) with psychotherapy (such as cognitive behavioral strategies, particularly stress management and problem-solving techniques) is especially important among MHO patients.

## Introduction

Young adulthood is a key life stage, and is when eating habits become established that can influence long-term health (1, 2). Poor nutritional patterns, characterized by high consumption of ultra-processed foods, sugary drinks, and fast food, along with a low intake of fresh, nutritious foods, are common in this population and are closely linked to increased overweight and obesity (3–5). The many contributing factors include a change in lifestyle when entering university, with students often having insufficient time to prepare healthy meals (6, 7). Furthermore, social pressure, anxiety, stress and lack of nutritional education are key factors that affect young people’s eating decisions, leading them to choose unhealthy foods as a means to manage emotions, thus contributing to weight gain (8–10).

Research indicates that overweight and obesity in young adults are linked to deficiencies in essential minerals and vitamins (11–13). The lack of micronutrients such as vitamins B and D, magnesium, and zinc can negatively impact metabolism, appetite, hormone function and gut microbiota regulation, ultimately increasing the risk of obesity (14–16). Moreover, diets high in simple carbohydrates (such as refined sugars and ultra-processed products), are directly associated with a greater risk of overweight and obesity (17–19) as they can cause spikes in glucose and insulin, which promote fat storage and insulin resistance. These carbohydrates also have a low satiety index, which can lead to excessive calorie consumption. In contrast, complex carbohydrates (such as whole grains and legumes) are digested more slowly, and thus promote satiety and ultimately weight regulation (20). Studies have also associated obesity to the high consumption of fats and sodium. The saturated fats that are prevalent in ultra-processed foods and red meats can increase the accumulation of abdominal fat and alter hormonal signaling (21, 22). Trans fats further disrupt appetite regulation, while excess sodium from over-reliance on processed and fast food can lead to fluid retention and impact metabolism, which also indirectly contribute to weight gain (18).

Studies have observed the influence of multiple sociodemographic variables on dietary patterns and hence metabolic status, giving rise to overweight and obesity. For example, women tend to be more concerned with body image and men more focused on increasing muscle mass (23–25), while unmarried individuals tend to consume more fast food, as opposed to the more organized eating patterns of married couples. Socioeconomic status is also a key factor, as young adults from lower strata have less access to healthy foods, while those from higher strata may be equally prone to choosing unhealthy options due to lack of time (25).

Overweight and obesity are related to excess body fat, which is primarily measured using the Body Mass Index (BMI). Overweight is defined as a BMI between 25 and 29.9, and obesity as 30 or higher (26). Both conditions raise the risk of cardiovascular disease, type 2 diabetes, hypertension (HTN), sleep apnea, joint problems, and certain types of cancer (27–29), with a distinction having been made between metabolically healthy obesity (MHO) and metabolically unhealthy obesity (MUO). Despite their excess body fat, individuals in the MHO group have normal or healthy glucose, insulin, cholesterol, and triacylglycerol levels, indicating a lower likelihood of developing metabolic diseases (30). In contrast, MUO subjects present high levels for these factors, and are therefore at a greater risk of developing diabetes, cardiovascular disease, and metabolic syndrome (31). Among young adults, physical activity also plays a crucial role in determining MHO versus MUO, and hence the risk of chronic diseases (32).

While the MHO–MUO distinction is analytically useful for characterizing heterogeneity within obesity, it is imperative to avoid misinterpreting MHO as a truly ‘healthy’ state. The absence of manifest metabolic disturbances in MHO could be interpreted as a limitation of current diagnostic thresholds rather than protection from risk. Excess adiposity itself remains a causal determinant of multiple adverse outcomes, including cardiometabolic disease, functional decline, and premature mortality. Therefore, treating MHO as benign risks obscuring the well-established long-term hazards of obesity. From both a methodological and public health standpoint, research and policy must continue to emphasize that excess adiposity, regardless of metabolic profile, constitutes a critical target for prevention and intervention (33).

Studies have also highlighted that stress, anxiety, and depression affect metabolic health in young adults (34, 35). Specifically, young MHO adults may experience all these issues, but they tend to demonstrate more efficient emotional regulation, which steadies their metabolic profile (36). However, among MUO, these emotional and psychological disorders are more prevalent and have a negative impact on metabolic health by promoting poor eating habits, and increasing cortisol (stress hormone) levels and fat accumulation, which worsens insulin resistance (37). Therefore, stress management and mental health are crucial matters that need to be addressed in order to prevent metabolic deterioration in young, obese adults (38).

In summary, investigation of the underlying associations between nutritional patterns, metabolic status, and psychological state of young adults is crucial because these factors are interconnected and affect long-term health (39). A better understanding of these networks in overweight/obese populations could be a useful springboard for developing interventions that can effectively enhance the quality of life of young adults. A more comprehensive approach would also benefit public health programs and long-term disease prevention.

The objective of this study was to apply network analysis to visualize the structure of interrelationships between dietary patterns and physical and psychological measures among young overweight/obese adults. We also sought to identify the nodes with the highest centrality indices and to explore their empirical modularity. Separate networks were developed for MHO and MUO individuals, to assess the potential moderator role of the metabolic state.

## Methods

### Participants

This study analyzed data recruited as part of a broader research project, aimed to identify mechanisms contributing to the cardiometabolic health status of young university students with overweight or obesity. The study of Reivan and colleagues contains the complete description of the sampling procedure (40).

The study included *N* = 188 young overweight and obese adults from the Catholic University of Cuenca (Azogues, Ecuador), who participated voluntarily and signed an informed consent form. Pregnant women, those with endocrine or genetic disorders, those on a weight-loss diet, or those taking supplements or medications that could affect their blood glucose, lipid profile, body weight, or blood pressure were excluded. Those with eating disorders were not excluded, as the objective was to obtain evidence on the young overweight or obese population in general, thus enhancing the external validity of the results.

### Measures

#### Anthropometry

BMI was the primary measure of nutritional status, calculated by dividing weight (kg) by height (m^2^). Following the World Health Organization (41), BMI was classified into two levels: overweight (25 ≤ BMI < 30) and obesity (BMI ≥ 30).

#### Dietary patterns

The FCFF questionnaire (42) was used to record dietary intake, with a nutritionist instructing participants to record the frequency and quantity of foods consumed over the past 12 months, based on standard portion sizes. Total energy and nutrient intake was calculated by summing the values for all foods, and the data were managed using Nutrimind software (available at https://www.nutrimind.net/). This study focused on the factor scores obtained from the FCFF, which were categorized into three distinct nutrient-based dietary patterns, following Reivan Ortiz et al. (40). The first pattern was labeled “NP1: high content of minerals and vitamins,” and measures the intake of potassium, magnesium, folate, pantothenic acid, riboflavin, phosphorus, zinc, calcium, vitamins B12, B6 and C, and fiber. The second pattern was labeled “NP2: high carbohydrate,” and characterizes the intake of thiamine, niacin, carbohydrates and iron. And the third pattern was labeled “NP3: high fat and sodium,” and assesses the intake of polyunsaturated, saturated and monounsaturated fatty acids, and also sodium.

#### Cardiovascular and metabolic risk factors

Anthropometric indices and cardiometabolic risk factors were measured by a nurse specializing in nutrition. Weight was measured using a calibrated electronic scale (BCS-G6), and height using a stadiometer, both with an accuracy of 0.1. Waist circumference was measured twice and the average was calculated (43). After a 5-min rest, two diastolic and systolic blood pressure measurements were taken 15 min apart on the right arm, and the average of both measurements was analyzed (44). Biochemical values were obtained from venous blood samples after a 12-h fast, measuring glucose, lipid profile, and insulin. Insulin resistance was calculated using the HOMA-IR model (45). Two methods are typically used to classify metabolic health: the first, based on modified International Diabetes Federation criteria (46), which classifies participants who have at least two risk factors as MUO, and the method based on the HOMA-IR index, derived from the following formula that integrates the levels of fasting glucose and insulin:


$$\text{HOMA}-\mathrm{IR}=(\begin{array}{l} \text{fasting glucose}\;[\mathrm{mg}/\mathrm{dL}]\;\times \;\\ \text{fasting insulin}\;[\mathrm{mU}/L]\; \end{array})/405$$

The formula when glucose is in mmol/L: (fasting glucose [mmol/L] × fasting insulin [mU/L] / 22.5).

This study employed the HOMA-IR criterion, a procedure frequently applied both in research and clinical settings for classifying obese individuals in the groups MUO (HOMA-IR ≥ 3) and MHO (HOMA-IR < 3) (47–49). This approach allows for a clear distinction between obesity phenotypes based on a standardized measure of the metabolic health and thus be able to compare the findings of this research with other results available in the literature.

#### Psychological characteristics

The Depression, Anxiety, and Stress Scale (DASS-21) (50) was used to assess psychological status, using a 21-item self-report questionnaire employing a Likert scale ranging from 0 (never) to 3 (almost always). This scale was designed to measure these symptoms over the past week and provides a total score reflecting general psychological distress. In this study, internal consistency was high, with alpha coefficients of 0.80 for depression, 0.82 for anxiety, 0.86 for stress, and 0.90 for the total score.

#### Socioeconomic factors

Participants also reported other variables, such as age, sex, marital status, and socioeconomic status (SES), the latter based on the Hollingshead Four-Factor Index (51).

### Procedure

The sample recruitment procedure was approved by the Human Research Ethics Committee (CEISH) at the Catholic University of Cuenca, endorsed by the Ecuadorian Ministry of Public Health and the institution’s legal authorities, as well as the program supervisors. Signed consent was obtained from all subjects involved in the study.

An invitation was sent to potential candidates via institutional email. Accepted students participated in two assessment sessions. The first was held in the classroom, where two clinical psychologists and a nutritionist (two researchers from our team and trained in psychometric assessment) asked participants to complete questionnaires on nutritional, psychological, and sociodemographic data; these researchers guarantee that participants properly completed the measurement instruments and the absence of missing values. This lasted approximately 40 min. Participants were then informed of the date and time of the second session, during which a nurse and a clinical psychologist recorded anthropometric and biochemical data. All data were collected in October 2023. Participation was voluntary, with no financial or academic incentives.

### Data analysis

The analysis was performed with Gephi 0.9.2 for Windows (52), open-source multiplatform software (available at http://gephi.org) for exploring and visualizing networks. Network analysis is a methodological approach that conceptualizes a set of variables as a system of interconnected nodes, where edges represent the statistical associations between them. This approach has become increasingly common in psychopathology research, with many theoretical and methodological developments quickly gaining traction because of its capacity to holistically address the complexity of the multifaceted nature of mental disorders (53–55). In contrast to traditional multivariate techniques (e.g., regression, factor analysis, structural equation modeling), which typically assume that observed variables reflect latent constructs and impose a unidirectional structure of associations, network analysis offers a framework that is particularly well suited to our aims. By focusing on how elements interact with each other rather than reducing them to underlying dimensions, network analysis provides a more nuanced and dynamic representation of the phenomenon under study (56, 57). This approach allows us to estimate direct relationships among variables without relying on latent factors, to identify the most central or influential nodes within the system, and to capture the overall structure of interconnections.

Separate networks were obtained for participants with MHO and MUO. The nodes included in the analyses measured different constructs concerning the physical domain (BMI, HTN, glucose, insulin, cholesterol-T, triacylglycerol, and HOMA-index), psychological functioning (depression, anxiety and stress), and the three empirical nutritional patterns (NP1, NP2, NP3). Data structuring resulted in 13 nodes and 78 potential edges. Due to the cross-sectional data analyzed in this work, undirected network analysis was conducted (the edges [connections] between the nodes [variables] were bidirectional). Partial correlations (Rp) were defined by the weights of these edges, the aim being to assess the strength and direction of the relationships between each pair of nodes while controlling/adjusting for the other nodes in the structure. Since most of the edges had low weights (partial correlations close to 0, suggesting poor/null association), only those with Rp values of *p* < 0.25 were selected. Regarding this threshold, it must be noted that while the conventional threshold of *p* < 0.05 is widely used for conventional statistical analysis procedures, in some contexts (such as network modeling or preliminary exploratory multiple regression) using a higher threshold helps avoid prematurely excluding potentially relevant associations: this approach ensures that variables which may not reach conventional significance individually but could be important in the context of the full network are retained for consideration, enhancing the comprehensiveness and interpretability of the model.

Network characterization in this study was based on the range of statistics displayed by Gephi, specifically concentrating on eigenvector, betweenness and closeness centrality. Eigenvector centrality measures the global relevance of each node in the network, considering the weighted sum of all the degrees (weights) of the nodes to which it connects. This index reflects how well a node is connected to other highly connected nodes, meaning that a node’s influence increases if it is linked to influential neighbors. For example, in obesity-related networks, *emotional eating* often emerges with high eigenvector centrality. This is because it tends to be directly connected to multiple other relevant factors, such as *binge eating*, *food craving*, *depressive mood*, and *stress*, while also being linked to nodes that are themselves highly interconnected. This position indicates that emotional eating is not only influential on its own but also embedded within a cluster of highly connected behaviors and psychological factors. As a result, changes in emotional eating may reverberate widely across the network, potentially affecting both eating behaviors and associated psychological symptoms.

Betweenness centrality is a measure of the capacity of a node to connect different areas of the network, calculated as the number of times it appears on the shortest path between other pairs of nodes. Node/s with the highest betweenness value/s are considered “bridge” elements, as other nodes are highly dependent on their strong capacity for connecting different subareas of the network and transferring information. If a bridge node is removed, the whole network could collapse. Bridge metrics identify nodes that directly tie together different subnetworks, making them critical for understanding how activation can “spread” from one domain to another. For example, in obesity-related networks, *body dissatisfaction* often functions as a *bridge* between different symptoms or behavior communities. For instance, it may connect a cluster of psychological factors (e.g., low self-esteem, depressive mood, anxiety) with a cluster of eating behaviors (e.g., emotional eating, binge eating, dietary restraint). High bridge centrality indicates that body dissatisfaction plays a key role in linking otherwise separate domains, facilitating the spread of influence or activation from psychological vulnerabilities to eating behaviors, and vice versa. Targeting such bridge nodes could therefore be especially effective in interventions, as changes in these nodes may reduce comorbidity or prevent the reinforcement of maladaptive behaviors across clusters.

Meanwhile, closeness centrality measures how close a node is to all the other nodes in the network, based on the average distance between vertices in the graph. The higher the closeness centrality, the greater the independence of a given node from potential intermediary/control nodes (since the number of edges connecting it to other nodes is low). Nodes with high closeness can quickly interact with or influence the rest of the network. For example, in obesity-related networks, *physical inactivity* often exhibits high closeness centrality. This means it is, on average, relatively close to all other nodes in the network, such as *sedentary behavior*, *poor dietary habits*, *emotional eating*, and *low self-esteem*. A node with high closeness can quickly influence—or be influenced by—many other factors in the network. In practical terms, targeting physical inactivity may rapidly affect multiple interconnected behaviors and psychological factors, making it a strategically important symptom or behavior for interventions aiming to modify the broader obesity-related network.

Beyond centrality, we also sought to identify node communalities (also called node modules or clusters) (58), which group nodes that are highly interconnected to each other and relatively unconnected to nodes outside of their cluster.

Global measures were also obtained (59), namely average path length, diameter and density. Average path length reflects how efficiently information is transported within the network, and is obtained as the mean of the shortest paths between all pairs of nodes. Diameter measures the greatest distance between the two outermost nodes in the network and is obtained as the maximum eccentricity of any vertex in the graph. Finally, density measures how close the network is to completion, and is obtained as the ratio between the number of edges modeled compared to the total number of potential edges.

## Results

### Characteristics of the sample

The upper part of Table 1 presents the descriptives for the sociodemographic variables. Most participants in the study were men (56.9%) and single (78.7%). The social status index was distributed as follows: 37.8% low level, 33.0% mean level, and 29.3% high level. Mean age was 20.8 years (SD = 2.57) and mean BMI was 28.4 kg/m2 (SD = 2.9). The remaining variables included in the network are described in the lower part of Table 1.

**Table 1:** Descriptive for the sample.

Evaluated variables		Total sample *(N = 188)*		Subsample MHO *(N = 132)*		Subsample MUO *(N = 56)*		
		*n*	*%*	*n*	*%*	*n*	*%*	*p*
Sex	Women	81	43.1%	59	44.7%	22	39.3%	0.493
	Men	107	56.9%	73	55.3%	34	60.7%	
Marital	Single	148	78.7%	105	79.5%	43	76.8%	0.739
	Married—couple	35	18.6%	23	17.4%	12	21.4%	
	Divorced—separated	5	2.7%	4	3.0%	1	1.8%	
Social	Low	71	37.8%	46	34.8%	25	44.6%	0.436
	Mean	62	33.0%	45	34.1%	17	30.4%	
	High	55	29.3%	41	31.1%	14	25.0%	
		*Mean*	*SD*	*Mean*	*SD*	*Mean*	*SD*	
Age (years-old)		20.76	2.57	20.62	2.47	21.09	2.79	0.254
BMI (kg/m^2^)		28.36	2.94	27.63	2.28	30.10	3.56	<0.0001
Nutritional pattern 1 (NP1)		7066.55	1883.53	7249.85	1905.83	6634.49	1772.06	0.040
Nutritional pattern 2 (NP2)		271.37	45.46	271.83	45.21	270.28	46.44	0.831
Nutritional pattern 3 (NP3)		77.05	18.36	76.63	18.49	78.05	18.16	0.628
Depression		98.89	11.20	94.29	5.45	109.72	13.61	<0.0001
Anxiety		10.41	2.93	9.34	2.42	12.90	2.47	<0.0001
Stress		193.29	52.11	179.67	39.20	225.40	63.85	<0.0001
Glucose		147.80	52.43	139.03	49.56	168.46	53.62	<0.0001
Insulin		2.54	0.78	2.16	0.55	3.44	0.44	<0.0001
Cholesterol-T		5.89	1.30	5.74	1.27	6.23	1.31	0.018
Triacylglycerol (TAG)		5.21	1.55	5.08	1.54	5.54	1.54	0.062
HOMA-IR index		10.69	2.62	10.46	2.60	11.23	2.61	0.065
		*n*	*%*	*n*	*%*	*n*	*%*	
Hypertension	No	140	74.5%	113	85.6%	27	48.2%	<0.0001
	Yes	48	25.5%	19	14.4%	29	51.8%	

MHO, metabolic healthy obesity; MUO, metabolic unhealthy obesity; BMI, body mass index; HOMA-IR, Homeostasis Model Assessment Insulin Resistance; NP1, minerals and vitamins; NP2, carbohydrates; NP3, fat and sodium.

Comparison between the MHO versus MUO groups reveals no differences in terms of socidemographic variables, nor in the mean factor scores for NP2 (carbohydrates), NP3 (fat and sodium), TAG, or HOMA-IR index.

### Network analysis of MHO subsample

**Figure 1:**
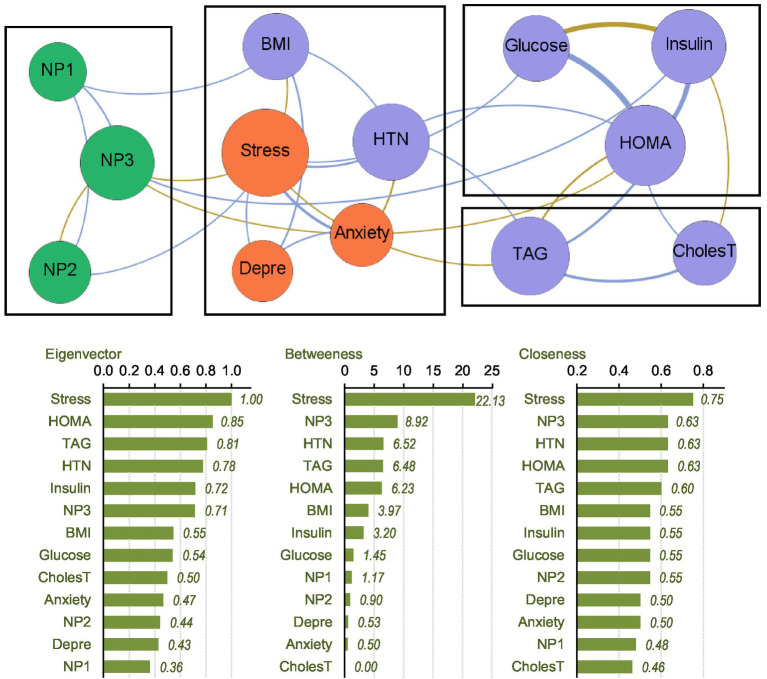
Visualization of the network among the MHO subsample. Positive edges are represented by blue lines, and negative edges by brown-ochre lines. As thicker the edge as stronger the connection weight. Nodes are plotted in colors depending on the dimension: nutritional patterns (green), physical variables (purple), psychological variables (orange). Nodes are identified as: NP1 (nutritional pattern 1, minerals and vitamins), NP2 (nutritional pattern 2, carbohydrates), NP3 (nutritional pattern 3, fat and sodium), BMI (body mass index), HTN (hypertension), Glucose, Insulin, CholesT (cholesterol total), TAG (triacylglycerol), HOMA (HOMA-IR index), Depre (depression), Anxiety, Stress. Clusters of nodes are grouped inside boxes.

The upper part of Figure 1 presents the network-graph for the MHO subsample (see Table 2 for complete network parameters). Nodes are color-coded by dimension: nutritional patterns (green), physical variables (purple) and psychological variables (orange). Edges with positive weights are shown as blue lines (Rp > 0, positive relationship between nodes), and negative weights as ochre /brown lines (Rp < 0, negative relationship between nodes). A total of 28 edges were selected for analysis, resulting in a network density of 0.359, diameter of 3, and average path length of 1.79.

**Table 2:** Results of the network within subsamples of MHO and MUO individuals.

Label	Dimension	ID	Closeness centrality	Harmonic closeness centrality	Betweeness centrality	Authority	HUB	Modularity	Clustering. coefficient	Number triangles	Eigenvector centrality
MHO subsample											
Body mass index	Physical variables	BMI	0.5455	0.6389	3.9667	0.2274	0.2274	1	0.3333	2	0.5456
Hyperthension	Physical variables	HTN	0.6316	0.7083	6.5167	0.3280	0.3280	1	0.4000	4	0.7751
Glucose	Physical variables	Glucose	0.5455	0.6111	1.4500	0.2307	0.2308	2	0.3333	1	0.5404
Insulin	Physical variables	Insulin	0.5455	0.6667	3.2000	0.3077	0.3077	2	0.5000	5	0.7167
Cholesterol-total	Physical variables	CholesT	0.4615	0.5556	0.0000	0.2149	0.2149	3	1.0000	3	0.4976
Triacylglycerol	Physical variables	TAG	0.6000	0.6944	6.4833	0.3454	0.3454	3	0.5000	5	0.8077
HOMA-IR index	Physical variables	HOMA	0.6316	0.7361	6.2333	0.3649	0.3649	2	0.4000	6	0.8517
Depression	Psychological state	Depre	0.5000	0.5833	0.5333	0.1781	0.1781	1	0.6667	2	0.4278
Anxiety	Psychological state	Anxiety	0.5000	0.5833	0.5000	0.1956	0.1956	1	0.6667	2	0.4666
Stress	Psychological state	Stress	0.7500	0.8333	22.1333	0.4204	0.4203	1	0.2143	6	1.0000
NP1	Nutritional patterns	NP1	0.4800	0.5694	1.1667	0.1505	0.1505	4	0.3333	1	0.3633
NP2	Nutritional patterns	NP2	0.5455	0.6111	0.9000	0.1842	0.1842	4	0.6667	2	0.4423
NP3	Nutritional patterns	NP3	0.6316	0.7083	8.9167	0.3014	0.3014	4	0.3000	3	0.7145
MUO subsample											
Body mass index	Physical variables	BMI	0.6667	0.7500	4.1667	0.2977	0.2977	2	0.4000	6	0.7988
Hyperthension	Physical variables	HTN	0.6000	0.6667	2.6667	0.1764	0.1764	2	0.3333	2	0.4799
Glucose	Physical variables	Glucose	0.6000	0.6944	0.2500	0.3054	0.3054	1	0.9000	9	0.7838
Insulin	Physical variables	Insulin	0.6000	0.6944	0.2500	0.3054	0.3054	1	0.9000	9	0.7838
Cholesterol-total	Physical variables	CholesT	0.7059	0.7917	7.3333	0.3824	0.3824	1	0.5238	11	0.9927
Triacylglycerol	Physical variables	TAG	0.6667	0.7500	8.2500	0.3000	0.3000	1	0.4667	7	0.7787
HOMA-IR index	Physical variables	HOMA	0.6000	0.6944	0.2500	0.3054	0.3054	1	0.9000	9	0.7838
Depression	Psychological state	Depre	0.6000	0.6667	3.1667	0.1700	0.1700	2	0.1667	1	0.4616
Anxiety	Psychological state	Anxiety	0.5217	0.6250	1.5000	0.1583	0.1583	2	0.3333	2	0.4386
Stress	Psychological state	Stress	0.6000	0.6667	2.3333	0.2010	0.2010	2	0.5000	3	0.5426
NP1	Nutritional patterns	NP1	0.6667	0.7500	4.3333	0.2641	0.2641	2	0.4667	7	0.7191
NP2	Nutritional patterns	NP2	0.6316	0.7083	2.6667	0.2331	0.2331	2	0.5000	5	0.6344
NP3	Nutritional patterns	NP3	0.7059	0.7917	9.8333	0.3818	0.3818	1	0.4762	10	1.0000

MHO, metabolic healthy obesity; MUO, metabolic unhealthy obesity; BMI, body mass index; HOMA-IR, Homeostasis Model Assessment Insulin Resistance; NP1, minerals and vitamins; NP2, carbohydrates; NP3, fat and sodium.

Four modules were identified in the network (shown in boxes in Figure 1). The three nutritional patterns were clustered within a single module. The psychological variables were grouped with BMI and the presence of HTN. The HOMA-IR index, and glucose and insulin levels were grouped in another cluster, while TAG level was grouped with cholesterol. The average clustering coefficient was 0.486.

**Figure 2:**
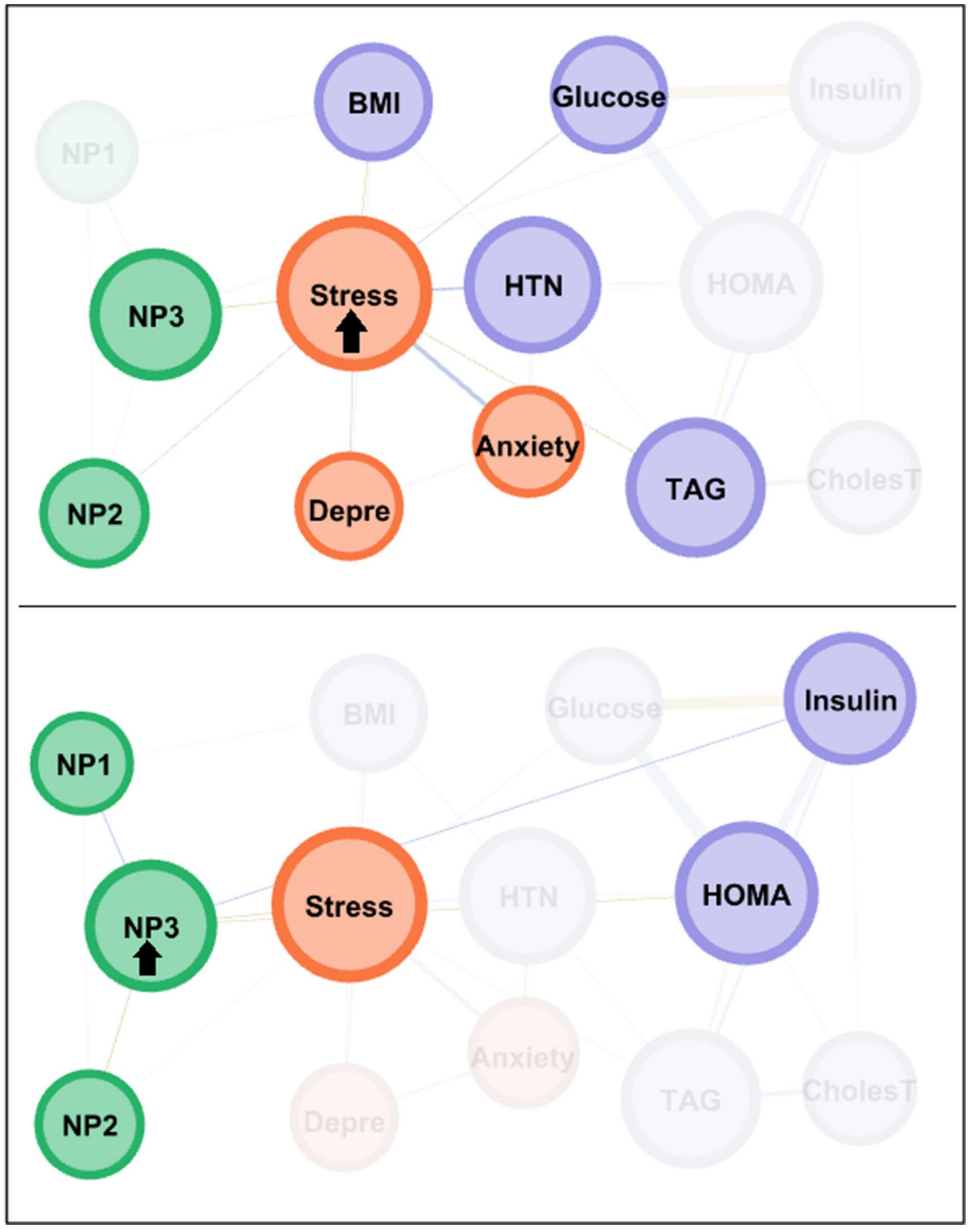
Main connections of the nodes with the highest centrality among the MHO subsample. Positive edges are represented by blue lines, and negative edges by brown-ochre lines. As thicker the edge as stronger the connection weight. Nodes are plotted in colors depending on the dimension: nutritional patterns (green), physical variables (purple), psychological variables (orange). Nodes are identified as: NP1 (nutritional pattern 1, minerals and vitamins), NP2 (nutritional pattern 2, carbohydrates), NP3 (nutritional pattern 3, fat and sodium), BMI (body mass index), HTN (hypertension), Glucose, Insulin, CholesT (cholesterol total), TAG (triacylglycerol), HOMA (HOMA-IR index), Depre (depression), Anxiety, Stress. Clusters of nodes are grouped inside boxes.

The centrality coefficients are shown in the lower part of Figure 1 (with nodes ranked from the largest to lowest centrality using bar-charts). The node for perceived stress level had the highest centrality across all three metrics (eigenvector, betweenness and closeness measures) and was hence the bridge node (due to having the highest capacity for connecting other nodes and transferring information throughout the whole structure). NP3 (fat and sodium) also presented high centrality, particularly in terms of betweenness and closeness. Figure 2 shows the main connections for these two most central nodes (stress and NP3). Activation of the stress node had a strong impact on depression, anxiety, and the other two nutritional patterns (NP2 and NP3). Activation of the NP3 node had a strong impact on the other two nutritional patterns, stress, insulin and HOMA-IR.

### Network analysis of the MUO subsample

**Figure 3:**
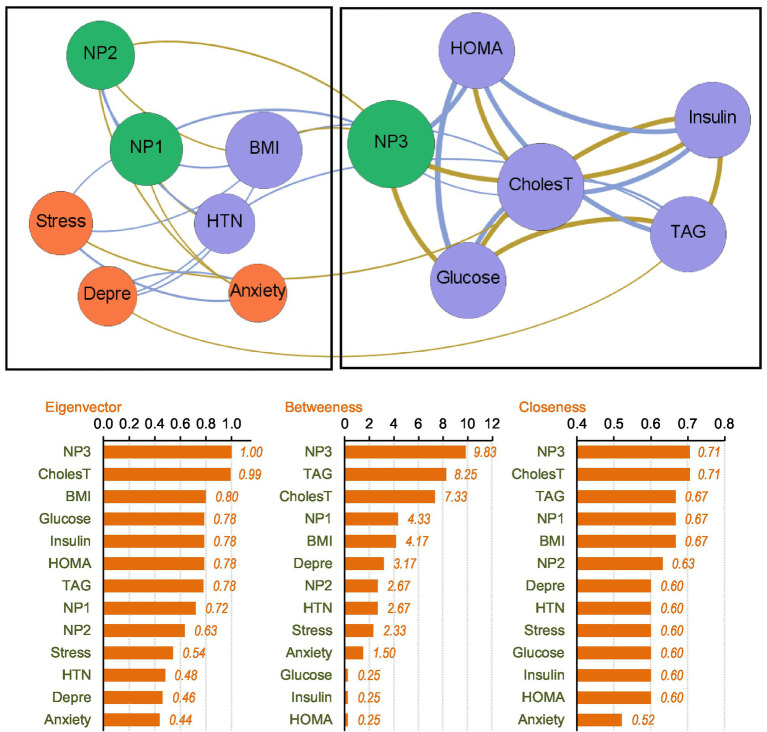
Visualization of the network among the MUO subsample. Positive edges are represented by blue lines, and negative edges by brown-ochre lines. As thicker the edge as stronger the connection weight. Nodes are plotted in colors depending on the dimension: nutritional patterns (green), physical variables (purple), psychological variables (orange). Nodes are identified as: NP1 (nutritional pattern 1, minerals and vitamins), NP2 (nutritional pattern 2, carbohydrates), NP3 (nutritional pattern 3, fat and sodium), BMI (body mass index), HTN (hypertension), Glucose, Insulin, CholesT (cholesterol total), TAG (triacylglycerol), HOMA (HOMA-IR index), Depre (depression), Anxiety, Stress. Clusters of nodes are grouped inside boxes.

The upper part of Figure 3 presents the network-graph for the MUO subsample (see Table 2 for the complete parameters). Thirty-four edges were selected for analysis, and network density was 0.436, diameter was 3, and average path length was 1.60.

Two empirical clusters of nodes were identified. The first grouped NP1 (minerals and vitamins), NP2 (carbohydrates), BMI, HTN and psychological state. The second module clustered NP3 (fat and sodium), HOMA-IR index, cholesterol, glucose, insulin and TAG. The average clustering coefficient was 0.528.

**Figure 4:**
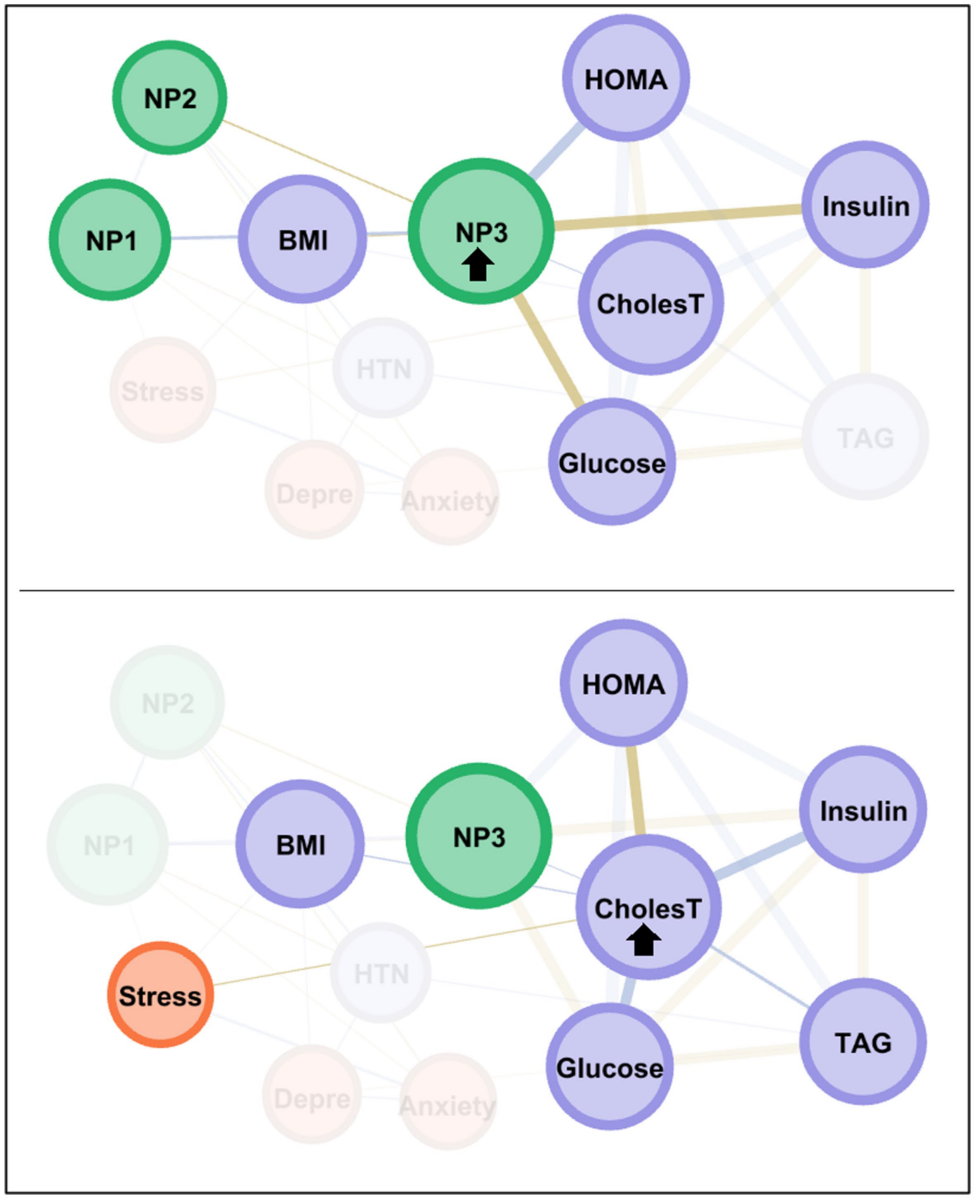
Main connections of the nodes with the highest centrality among the MUO subsample. Positive edges are represented by blue lines, and negative edges by brown-ochre lines. As thicker the edge as stronger the connection weight. Nodes are plotted in colors depending on the dimension: nutritional patterns (green), physical variables (purple), psychological variables (orange). Nodes are identified as: NP1 (nutritional pattern 1, minerals and vitamins), NP2 (nutritional pattern 2, carbohydrates), NP3 (nutritional pattern 3, fat and sodium), BMI (body mass index), HTN (hypertension), Glucose, Insulin, CholesT (cholesterol total), TAG (triacylglycerol), HOMA (HOMA-IR index), Depre (depression), Anxiety, Stress. Clusters of nodes are grouped inside boxes.

The node with the highest centrality was NP3, as shown in the bar charts in the lower part of Figure 3. The node measuring cholesterol level also presented very high coefficients for eigenvector and closeness. Figure 4 illustrates the main connections for these two most central nodes (NP3 and cholesterol). Activation of the NP3 node had a high impact on the other nutritional patterns (NP1 and NP2), as well as most nodes measuring physical profile (BMI, HOMA-IR, cholesterol, glucose and insulin). Activation of the cholesterol node had a high impact on the remaining physical measures (except for HTN), as well as on NP3 and stress.

## Discussion

The primary objective of this study was to apply network analysis to visualize the structure of interrelationships between nodes representing nutritional patterns, metabolic measures, and psychological health in young overweight adults with and without cardio-metabolic risk factors. Network analysis identified the nodes with the highest centrality and their main connectivity within each subsample, providing a comprehensive view of the factors contributing to the differences between these two obesity groups.

No significant differences were found between the MHO and MUO groups in terms of sociodemographic variables and some metabolic and nutritional measures (such as triacylglycerol levels and HOMA-IR index). This seems to suggest that sociodemographic characteristics alone are insufficient to discriminate between MHO vs. MUO, and instead there is a far more complex network of relationships between a wide variety of domains and constructs. Previous studies support this view, indicating that obesity and metabolic diseases are multifactorial conditions that depend on a dynamic interplay between genetic, biological, environmental, and behavioral factors (60–63). Hence, although age, sex, socioeconomic status and ethnicity might all somehow influence the risk of developing metabolic diseases (64, 65), none of these factors fully explain why some obese people remain metabolically healthy, while others develop complications such as diabetes and HTN. Other studies have posited that metabolic health is closely tied to body fat distribution, individual genetics, physical activity, eating habits, stress, inflammation, or insulin resistance, rather than sociodemographic characteristics alone (66–69).

### Network structure in MHO individuals

The MHO network presented a modular structure that grouped psychological variables with BMI and the presence of HTN in a single cluster, suggesting that psychological aspects such as stress may have a direct impact on physical health in this obesity type. This aligns with other studies that have suggested that chronic stress influences the physical health of these individuals due to prolonged activation of the hypothalamic–pituitary–adrenal (HPA) axis, which increases the release of the stress hormone cortisol (70, 71) and has thus been associated to a range of adverse physiological effects, including insulin resistance, systemic inflammation, and hormonal imbalance (72). Although MHO individuals may not present evident dysfunctions in metabolic parameters such as glucose or lipids, prolonged stress can impair endothelial function, promote immune system dysfunction, and increase the risk of cardiovascular disease (73, 74).

Likewise, in the MHO network, the stress node also presented the highest centrality in the dynamics between metabolic health and psychological and nutritional factors, highlighting its important role as a bridge connecting factors such as depression, anxiety, and eating behavior, and particularly NP3 (fats and sodium). Previous studies corroborate our results by sustaining that chronic stress can have a significant impact on these factors in MHO individuals driven by neuroendocrine and behavioral mechanisms (75–77). Prolonged activation of the endocrine stress response system elevates cortisol levels, which, in turn, impairs emotional regulation and neuronal plasticity, promoting anxiety, depression and other emotional difficulties, even in the absence of evident metabolic disorders (8). Similarly, studies also report that stress activates reward circuits in the brain, increasing the likelihood of eating foods rich in fat and sodium as a strategy to mitigate emotional distress, which can trigger a dopaminergic response that reinforces these behaviors (78, 79). In summary, in the context of obesity, stress and dietary factors are interconnected through both physiological and behavioral pathways. Physiologically, stress activates the hypothalamic–pituitary–adrenal axis, leading to elevated cortisol levels, which can increase appetite and preference for energy-dense foods (80, 81). Behaviorally, stress often triggers reward-driven or emotional eating, which may reinforce maladaptive eating patterns (82, 83). In a network perspective, nodes such as emotional eating and food cravings often occupy central or highly influential positions, reflecting their key role in linking psychological stress with dietary behaviors. Empirical evidence supports this connection: higher cortisol and perceived stress are associated with increased caloric intake and consumption of ultra-processed foods, highlighting mechanisms by which stress may contribute to obesity-related behaviors and outcomes.

Interesting, our findings align with emerging evidence indicating that young adults can experience cardiovascular events, such as myocardial infarction, despite the absence of classical metabolic abnormalities (such as obesity) (84, 85), often presenting only mild overweight and elevated stress levels (86). In the present study, stress emerged as the central bridge node in the MHO network, suggesting that the apparent ‘metabolic health’ of these individuals may conceal underlying vulnerabilities. Unlike routine metabolic biomarkers, chronic psychosocial stress is challenging to detect in standard clinical assessments, as it manifests through complex behavioral, psychological, and neuroendocrine pathways—such as poor sleep, elevated perceived stress, mood disturbances, increased cortisol, or autonomic dysregulation. Consequently, individuals classified as metabolically healthy may still carry substantial cardiovascular risk driven by persistent stress exposure. These observations underscore the value of incorporating multidimensional assessments into research and clinical practice, including validated psychological stress questionnaires, biomarkers like cortisol, heart rate variability, and structured evaluations of lifestyle factors, to identify hidden vulnerabilities and inform preventive interventions even when conventional metabolic indicators remain within normal ranges.

The node measuring NP3 (fats and sodium) also displayed high centrality in the MHO network. Activation of this node had a high impact on the other nutritional patterns (NP2 and NP1), insulin and HOMA-IR index, which could be related with the ability of young adults with MHO to maintain metabolic balance despite the presence of obesity by controlling their psychological state and dietary factors. Previous studies support these findings, suggesting that stress management and resilience are key protective factors for this group, while higher stress and depression levels can impair metabolic regulation (38, 87–89). It has also been observed that healthy diets rich in anti-inflammatory foods and fiber can improve metabolic function and prevent obesity-related diseases, even in the absence of significant weight loss (90).

### Network structure in MUO individuals

The MUO network featured two clusters of nodes. The first grouped NP1 (minerals and vitamins) and NP2 (carbohydrates), BMI, HTN, and psychological variables, while the second grouped NP3 (fats and sodium) and key physical indicators such as HOMA-IR, cholesterol, glucose, insulin, and triacylglycerol. The higher density in this network (compared to the MHO group) suggests the variables could be more interrelated among MUO individuals and that metabolic and nutritional factors play a more interdependent role. This may be because MUO patients often present nutritional imbalances, such as diets rich in saturated fats, sugars, and sodium, which contribute to metabolic disorders such as insulin resistance, dyslipidemia (high levels of LDL cholesterol and triglycerides), and chronic inflammation. Indeed, previous studies have found that these metabolic dysfunctions can lead to a poorer nutritional profile, creating a cycle in which poor diet intensifies metabolic problems and vice versa (91, 92). Therefore, despite the presence of high BMI among MHO individuals, these subjects exhibit better metabolic control, with normal levels of glucose, cholesterol, and blood pressure, suggesting that adequate nutritional factors and a healthy lifestyle may mitigate the negative effects of obesity. The potentially stronger interdependence between nutritional and metabolic factors among MUO individuals would therefore contribute to a more pronounced decline in health.

The NP3 node (fats and sodium) demonstrated the highest centrality in the MUO network, reflecting its importance in the etiology of this group. This dietary pattern seems highly conducive toward an unhealthy obesity profile, and directly influences metabolic and psychological health. Prior studies have also found that foods high in fat and sodium promote caloric excess and metabolic alterations, such as insulin resistance and HTN, which in turn increase the risk of diseases like type 2 diabetes (93, 94). These foods can also impact brain chemistry, which has been associated to increased anxiety, stress, and depression, creating a vicious cycle of emotional eating and psychological distress (95–97). These components have also been observed to disrupt the gut microbiome, leading to nutritional deficiencies that worsen both physical and emotional health, perpetuating the negative effects on the body (98).

The cholesterol node was also highly central in the MUO group, suggesting a close link to nutritional patterns and the presence of comorbidities associated with obesity, such as HTN. This aligns with claims in the literature that cholesterol is closely related to dietary profile and comorbid conditions related to obesity, such as high blood pressure, because diets high in saturated and trans fats elevate LDL (bad) cholesterol levels, promoting atherosclerosis and reducing blood flow (99, 100). Obesity, which has been strongly linked to insulin resistance and chronic inflammation, leads to dysfunctional lipid metabolism, thus increasing LDL cholesterol and triglyceride levels, while reducing HDL (good) cholesterol (101). These lipid imbalances have also been associated to the onset and progression of HTN, since the accumulation of fat in the arteries hinders blood flow, raising blood pressure and increasing the risk of cardiovascular and metabolic diseases (101–103).

### Limitations

The findings of this study must be interpreted in light of certain limitations. First, this work analyzed cross-sectional data, resulting in undirected networks: the edges (connections) represented correlation-based associations between the nodes (variables), meaning that the relational patterns represented statistical relationships rather than causal effects. Further research with longitudinal designs should help to define the specific roles of the nodes as exposures or responses, and to test causal relationships between variables. Second, although the study included a set of nodes measuring different constructs, it was not possible to include other factors concerning endophenotype that may influence metabolic health status. Future research should raise the number of nodes and domains, to obtain a more complete picture of the obesity profiles. Third, the sample only included young adults, which limits generalization of the results to older populations, in whom the interrelationship between metabolically healthy and unhealthy obesity could follow different patterns. Similarly, it would be useful to expand this network analysis to samples with different sociodemographic characteristics (such as ethnicity, education and cultural background). Fourth, dietary data were self-reported, which makes them susceptible to recall bias: participants may not accurately remember or report their food intake, leading to potential misestimation of actual dietary behaviors, and this could affect the precision of associations between dietary factors and other variables in the study. And finally, because the sample was based on convenience rather than random selection, it cannot be assumed that the participants are fully representative of the broader population of young university students, which may limit the generalizability of the findings. Even, there may be potential variables not measured or analyzed in this study that could act as confounding factors, which might influence the networks potentially biasing the observed associations.

### Strengths

This study provides valuable insight into the interrelationships between physical, nutritional, and psychological variables in young overweight or obese adults, and furthers our understanding of the complex networks underlying metabolically healthy and unhealthy obesity. The results suggest that psychological stress and nutritional patterns are key factors in distinguishing between MHO and MUO, and this observation may have implications for obesity prevention and treatment. In particular, the centrality of stress in the MHO group highlights the importance of addressing psychological factors when addressing metabolically healthy obesity, as these could play a crucial role in maintaining a healthy metabolic profile despite obesity. In the MUO group, the high centrality of NP3 and cholesterol suggests that dietary programs and the control of metabolic factors could be key to reducing the risk of comorbidities associated with unhealthy obesity.

This study also underscores the usefulness of network analysis as a tool to explore the interconnection between multiple factors influencing metabolic health. Unlike traditional multivariate methods, network analysis allows to examine direct interactions among variables, identify central and bridge nodes, and capture the overall structure of interrelations, providing a more dynamic understanding of factors contributing to obesity and its metabolic risk profiles. Furthermore, our findings have clear implications for future research and practice. Identifying central and bridge nodes in the network can guide longitudinal studies to track how these key factors influence the progression from MHO to MUO over time. Additionally, these insights could inform practical screening strategies, by highlighting which behaviors, psychological traits, or physiological measures may serve as early indicators of increased metabolic risk, enabling targeted interventions to prevent MUO transitions.

## Conclusion

Young adulthood (typically from ages 18 to 25) is a critical period for development because long-term lifestyle habits are often established during this transitional phase (104), when individuals are especially vulnerable to the onset and progression of obesity and metabolic diseases (105, 106). Research indicates that during this period of life, changes in diet, a more sedentary lifestyle, and lack of time for exercise are all potential contributors to the adoption of unhealthy behaviors that increase the risk of type 2 diabetes and cardiovascular diseases (107, 108).

Among young adults, overweight and obesity are strong predictors of adverse effects and an increased risk of developing multiple chronic diseases at older ages, including physical disorders (such as cardiovascular issues, HTN, diabetes, asthma, and bone diseases) and psychological issues (including anxiety, depression and sleep problems). The identification of key variables in patients with and without cardio-metabolic risk factors will contribute to the design of more precise, individualized intervention programs, aimed at promoting healthy habits. It is crucial to recognize that even in the absence of overt metabolic abnormalities, obesity confers a substantially increased risk of cardiometabolic disease and mortality. Clinicians should therefore avoid interpreting MHO as a harmless state: excess adiposity itself remains a critical determinant of long-term health risks. Public health strategies must therefore continue to prioritize the prevention and reduction of obesity in all its forms.

## Data Availability

The raw data supporting the conclusions of this article will be made available by the authors, without undue reservation.
